# An electronic health record-enabled obesity database

**DOI:** 10.1186/1472-6947-12-45

**Published:** 2012-05-28

**Authors:** G Craig Wood, Xin Chu, Christina Manney, William Strodel, Anthony Petrick, Jon Gabrielsen, Jamie Seiler, David Carey, George Argyropoulos, Peter Benotti, Christopher D Still, Glenn S Gerhard

**Affiliations:** 1 Geisinger Obesity Research Institute, Geisinger Clinic, Danville, PA, 17822, USA; 2 Center for Health Research, Danville, PA, 17822, USA; 3 Weis Center for Research, Geisinger Clinic, Danville, PA, 17822, USA; 4 Department of Surgery, Geisinger Medical Center, Danville, PA, 17822, USA; 5 Department of Surgery, St. Francis Medical Center, Trenton, NJ, USA

**Keywords:** EHR, Database, Weight loss, Modeling, Obesity

## Abstract

**Background:**

The effectiveness of weight loss therapies is commonly measured using body mass index and other obesity-related variables. Although these data are often stored in electronic health records (EHRs) and potentially very accessible, few studies on obesity and weight loss have used data derived from EHRs. We developed processes for obtaining data from the EHR in order to construct a database on patients undergoing Roux-en-Y gastric bypass (RYGB) surgery.

**Methods:**

Clinical data obtained as part of standard of care in a bariatric surgery program at an integrated health delivery system were extracted from the EHR and deposited into a data warehouse. Data files were extracted, cleaned, and stored in research datasets. To illustrate the utility of the data, Kaplan-Meier analysis was used to estimate length of post-operative follow-up.

**Results:**

Demographic, laboratory, medication, co-morbidity, and survey data were obtained from 2028 patients who had undergone RYGB at the same institution since 2004. Pre-and post-operative diagnostic and prescribing information were available on all patients, while survey laboratory data were available on a majority of patients. The number of patients with post-operative laboratory test results varied by test. Based on Kaplan-Meier estimates, over 74% of patients had post-operative weight data available at 4 years.

**Conclusion:**

A variety of EHR-derived data related to obesity can be efficiently obtained and used to study important outcomes following RYGB.

## Background

Obesity is a multi-factorial disease that has reached epidemic proportions in the US, with about two thirds of adult Americans overweight (body mass index [BMI] > 25 kg/m^2^), one third obese (BMI >30 kg/m^2^), and about 5% with class III levels of obesity (BMI > 40 kg/m^2^) [1]. Obesity is associated with increased risk for a variety of co-morbidities, higher lifetime health care expenditures, and a greater risk for mortality [2]. Increasing BMI is associated with further increases in disease burden and risk of mortality [3] and the prevalences of both class III (BMI > 40 kg/m^2^) and super (i.e., BMI >50 kg/m^2^) obesity are rising faster than other classes of obesity [4]. Dietary modification and physical activity are effective at decreasing obesity-related disease risk and severity [5], although only modest weight loss is usually achieved and few individuals are able to maintain weight loss over long periods. In contrast, Roux-en-Y gastric bypass (RYGB) surgery commonly results in a more substantial and sustained weight loss, and has thus emerged as a more effective therapy for long-term weight loss in morbidly obese patients [6], and more recently, as a surgical therapy for type 2 diabetes [7]. However, the degree of weight loss with RYGB is variable [8,9] and the factors determining long-term success are not known. The effect of RYGB of various clinical outcomes is also not well characterized.

To conduct research on RYGB outcomes, significant resources are required for the long-term follow-up of patients as well as for clinical characterization. Economic efficiencies can be gained through leveraging existing clinical infrastructure. One such resource that has only recently been exploited for research is the electronic health record (EHR). Body mass index, calculated from height and weight, is perhaps the most commonly used measure of obesity and is often measured during clinical encounters and entered into EHRs. A large number of other variables, such as laboratory test results, the presence of co-morbid conditions, and medication use are also often recorded in EHRs. The potential availability of such data suggests that EHRs may be a rich resource for RYGB and obesity research.

We have developed a research database within the context of a Weight Management Clinic at a large integrated health system through utilization of an EHR fed data warehouse. A large number of clinical variables on patients undergoing RYGB surgery were extracted and assembled into research datasets. To illustrate the potential utility of the data, we estimated the length of post-operative follow-up after RYGB surgery. The efficient electronic acquisition of these data serves as a new paradigm for RYGB and obesity research.

## Methods

### Patients

**Figure 1:**
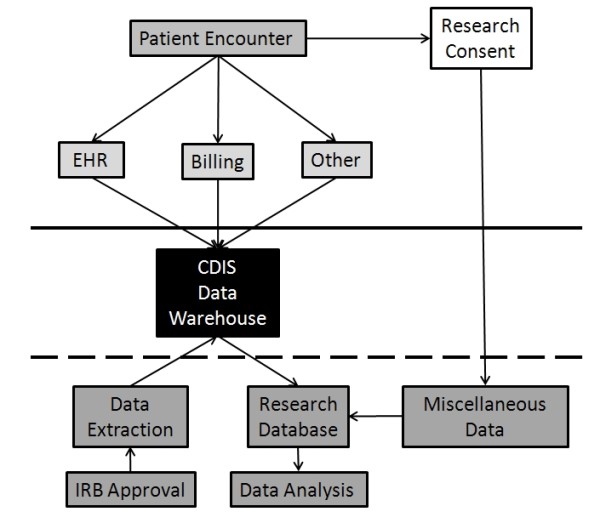
Flow diagram of data used to generate research database.

All patients who were enrolled in the bariatric surgery program in the Center for Nutrition and Weight Management at Geisinger Clinic were offered participation in an ongoing research program in obesity using clinical data accessed through the electronic health record that was approved by the Geisinger Clinic Institutional Review Board. For this study, a total of 2028 patients who underwent RYGB gastric bypass surgery from 01/01/2004 through 07/02/2010 were included in the database. The bariatric surgery program consisted of a 6 to 12 month pre-operative assessment and preparation period that included a diet-induced weight loss target of 10% of body weight. Patients were followed at approximately 1, 3, 5, and 12 months following RYGB surgery and every 12 months thereafter. All clinical data were entered into the EpicCare® EHR (Verona, WI). The EpicCare® EHR integrates information from a variety of sources into a common interoperable database that includes patient demographics, vitals, clinical measures, problem list (based on ICD-9 codes), medical history, medication history, personal and family histories, encounters (e.g. office visits, hospitalizations, nurse encounters, telephone inquiries and specialty consultations), orders (e.g. labs, medications, imaging and procedures), appointments, digital imaging (e.g. MRI, CT, X-ray, medical photography), results (e.g. procedure reports, lab results, pathology reports), and billing and claims databases (detailed financial transactions associated with each clinical encounter). All data except laboratory results, which were fed directly to the EHR by the laboratory information system, were entered at the point-of-care including age, sex, height, and weight, lifestyle factors (e.g., smoking, alcohol, etc.), clinical measures (e.g., blood pressure), all orders (i.e., lab requests, prescriptions, imaging, and procedures) which require at least one indication (i.e., ICD-9 code), active use of all medications, and all co-morbidities. The schema for data acquisition is shown in Figure 1.

### EHR and data warehouse

Data were extracted from an EHR-fed comprehensive enterprise-level data warehouse, the Clinical Decision Intelligence System (CDIS), which is partially comprised of the EHR. EpicCare EHR modules feeding data to CDIS included ambulatory, inpatient, surgery, emergency department, e-prescribing, computerized physician order entry, pharmacy, registration, scheduling, and reporting, thus data derived from clinical care provided not only at the Weight Management Clinic was included but also from other sites within the Geisinger Health System, including over 40 primary care sites and other specialty clinics. Other source systems, including financial decision support, insurance claims, patient satisfaction, and high-use third-party reference datasets were also deposited into CDIS.

CDIS was built on the IBM InfoSphere (DB2®) Warehouse 9 platform. The input data were extracted from several sources, predominantly the EpicCare EHR, and were transformed by selecting as needed by data type, and then loaded into the warehouse software by overwriting existing data with cumulative information. Clarity, an Oracle relational database with a data schema consisting of several thousand tables was used to report data out of EPIC using the extract-transform-load process. Extracts from Clarity consisted of preselected tables and data elements. These selected tables were transferred and loaded into CDIS using the IBM DB2 database software. The load into CDIS was then a data replication.

An analytics engine was built around the IBM Balanced Warehouse® for AIX® technology that enabled data mining, text analytics, reporting, and data analysis. In addition to the IBM InfoSphere Warehouse Information Server software and analytics engine, a reporting tool from IBM Business Partner Business Objects was used to provide user-friendly query and analysis, and data integration interface. The data in CDIS was stored as a relational database at the most granular level to allow for an effectively unlimited number of reporting, analysis, and application outputs. A copy of the CDIS data warehouse was stored on a separate server that allowed for direct data extraction into SAS using a Microsoft (Bellevue, WA) Open Database Connectivity (ODBC) interface. For example, the RYGB research datasets are refreshed on a regular basis (i.e. minimum every 3 months or sooner as needed). CDIS is updated on a nightly basis from EpicCare and less frequently from the other source systems (e.g., monthly).

### Data extracted

The extracted data for the RYGB database study include the following:

• Demographics: date of birth, gender, race, death status, and death date (one row per patient)

• Problem list: current and historical list of medical co-morbidities maintained and entered by treating providers (one row per diagnosis code per unit time)

• Outpatient office visit encounters: date of encounter, diagnoses assigned on that encounter, clinic that encounter took place, measurements taken at that encounter (e.g. weight, height, blood pressure, pulse, temperature) (one row per encounter)

• Inpatient admissions: admit and discharge dates, diagnosis codes, and name of admitting clinic (one row per admission).

• Medication list: current and historical list of active medications maintained and entered by treating providers (one row per diagnosis code per unit time)

• Medication prescription orders: date, name of medication, associated diagnosis for medication order, name of clinic that ordered medication (one row per medication order)

• Procedures: all inpatient and outpatient medical procedures including date, procedure code, and name of clinic that conducted procedure (one row per procedure)

• Laboratory test results: all laboratory results including dates, lab type, lab name, and lab resulted value (one row per laboratory result)

• Social history: historical and current alcohol and smoking history that are maintained and entered by treating providers (one row for each change in status over time)

• RYGB flowsheets: data collection tools that are entered for specific encounter types (e.g. RYGB surgical evaluation visit) that included but are not limited to dietician evaluation status, resting energy expenditure, surgeon, and weight loss goals

• Surveys: item responses to each survey question and date of survey completion

Most of the data were obtained as coded fields except for waist circumference, dietician evaluation status, psychologist evaluation status, resting energy expenditure, surgeon, tobacco and alcohol use at time of pre-surgical visit, and weight loss goals. These were free text but were recorded with pre-defined structures/guidelines that enabled consistent retrieval of the data. The extracted data were stored as SAS dataset files. Supporting data were gathered through departmental tracking databases (including landmark dates such as date of consent, date of initial visit, date of surgery, etc.) and through chart review (e.g. to validate the date and type of surgery).

### Survey data acquisition

At each new patient visit, the following surveys were obtained: Beck Depression Inventory (BDI) [10], Family Emotional Involvement and Criticism Scale (FEICS) [11], Impact Of Weight On Quality Of Life-Lite (IWQOL) [12], Weight Loss Readiness Test [13], Sleep Scale for Medical Outcomes [14], Work Limitations [15], and Questionnaire on Eating and Weight Patterns (QEWP) [16]. The Beck Depression Inventory and the Impact Of Weight On Quality of Life-Lite were initiated near the start of the program (i.e. patients with surgeries occurring since 10/01/2004) and were also administered during the post-operative period. The remaining surveys were administered to patients since 01/10/2006 but only in the pre-operative period.

Surveys were self-administered using one of two collection methods. The most common method was a paper survey formatted for electronic scanning using optical character recognition (OCR). These surveys were collected, batched for scanning using Kofax Capture software (Irvine, CA) and OCR processing using Sungard version 5.0 (Birmingham, AL), exported into delimited text files, and stored in SAS (Statistical Analysis System version 9.2, Cary, NC) datasets. The second method was an internet based EHR patient portal, MyGeisinger, allowing some patients to complete the surveys on-line. These on-line surveys were stored within the EHR and were available in the data warehouse described below. All survey responses were collected, imported into SAS, scored per validated algorithms (when applicable), and stored in SAS datasets.

### Dataset creation and cleaning

Datasets containing all of the clinical and survey data were created in SAS format, each containing a patient identifier used to link patient data between sources. SAS was used to manipulate, clean, and merge data for summary descriptive information and complex statistical analyses. Data cleaning algorithms were created to identify obvious errors and implausible values. An example of the algorithm used for weight and BMI is described below.

Clinically implausible values were identified and flagged for removal (e.g. weight <50 lbs. or weight >1000 lbs.). To identify other obvious data entry errors, i.e. those that fell within clinically acceptable ranges but were likely erroneous when compared to the patient’s other weight measures, a series of simple linear regression models (pre-surgery, 0–1 year post RYGB surgery, and 1+ years post RYGB surgery) were used. Due to the large number of weight measures for each patient, the linear models were run independently for each subject, and the distribution of residuals across all subjects was evaluated. Weight measures with residuals in the extreme tails of the distribution, i.e., +/− 5 standard deviations were flagged for consideration for exclusion. The flagged values were then manually evaluated and data entry errors were removed as indicated. BMI was calculated for all weight measures using the height recorded at the initial Weight management clinic visit. The presence of clinically implausible BMI values was investigated (e.g. BMI < 15 kg/m^2^ or BMI > 100 kg/m^2^) but none were found due to the initial weight cleaning algorithm.

### Statistical analysis

A descriptive summary was completed using means, standard deviation, and percentages, as appropriate. Kaplan-Meier curves were used to evaluate time until loss of follow-up. Patients were defined as lost to follow-up when they had a 24 month period without a weight measurement in the EHR. The date of surgery was used as the anchor point for the Kaplan Meier analysis. For those in active follow-up, the censor time was calculated by using date of last BMI measurement. The Kaplan Meier curves were stratified by initial BMI (35.0–39.9, 40.0–49.9, and 50.0+ kg/m^2^). The log rank test was used to evaluate whether length of follow-up was associated with initial BMI, year of surgery, surgeon, or surgical approach (open versus laparoscopic).

## Results

All patients enrolled in the Geisinger Bariatric Surgery clinical program are offered participation in the ongoing obesity research program. The results presented below were obtained from 2028 patients who had completed RYGB surgery between January 1, 2004 and July 2, 2010. The overall consent rate for this group was greater than 90%. The program continues to accrue patients at a similar rate.

### Demographic and weight data

The population was 81% female, 97% Caucasian ancestry, with a median age of 46 years (Table 1). Smoking and alcohol use data were not available on 8% and 13% of patients respectively, with a majority of the patients classified as either non-smokers or non-drinkers. Height, weight, and BMI were measured during initial weight management clinic visits. A median height of 65 cm and median weight of 292 pounds corresponded to a median BMI of 47.9 kg/m^2^. Median waist circumference was 52.8 cm at the initial clinical evaluation obtained on over 80% of patients. A total of 58 weight measurements (0.1% of 39,823 weight measures) from 42 patients (2% of 2028 patients) were classified as clinically implausible or data entry errors and were removed from the analysis.

**Table 1:** Demographic data (n=2028)

		
Gender		
	Male, n (%)	385 (19%)
	Female, n (%)	1643 (81%)
Age		
	Median [range]	46 [18, 72]
Race		
	White/Caucasian, n (%)	1960 (97%)
	Black, n (%)	44 (2%)
	Other, n (%)	24 (1%)
Smoking		
	Current, n (%)	204 (11%)
	Quit, n (%)	711 (38%)
	Never, n (%)	948 (51%)
	Not known, n (%)	165 (8%)
Any alcohol use		
	Yes, n (%)	729 (41%)
	No, n (%)	1029 (59%)
	Not known, n (%)	270 (13%)
Height (inches)		
	Mean (SD)	65.5 (3.4)
	Median [range]	65.0 [54.8, 78.0]
Weight (pounds)		
	Mean (SD)	303 (64)
	Median [range]	292 [180, 614]
BMI (kg/m2)		
	Mean (SD)	49.6 (8.8)
	Median [range]	47.9 [32.5, 94.3]
Waist circumference		
	Mean (SD)	53.5 (6.5)
	Median [range]	52.8 [39.0, 76.0]

### Co-morbid conditions

ICD-9 codes assigned during the pre-RYGB surgery period were used to identify the presence of co-morbid conditions. A total of 2067 unique 5 digit ICD-9 codes were found (Additional file 1: Table S1), with 177 occurring in at least 10% of the patients. Hypertension, hypercholesterolemia, diabetes, sleep disturbances (primarily sleep apnea), and depression were found in over one-third of the patients (Table 2). Diseases of the esophagus (primarily gastro-esophageal reflux disease or GERD), osteoarthrosis, hypothyroidism, and asthma were found in at least 15% of patients. The median number of ICD-9 diagnoses per patient was 12 with many patients having 20 or more (Additional file 1: Figure S1).

**Table 2:** Frequency of common co-morbidities (N=2028)

**Co-Morbidity**	**Frequency**
Hypertension	1147 (57%)
Hypercholesterolemia	881 (43%)
Diabetes	838 (41%)
Sleep disturbances	737 (36%)
Depression	717 (35%)
Diseases of esophagus	637 (31%)
Osteoarthrosis	534 (26%)
Hypothyroidism	369 (18%)
Asthma	314 (15%)

### Medication use

Patient medication use prior to surgery was obtained from the active medication list. A total of 349 medication subclasses were ordered (Additional file 1: Table S2), with 177 occurring in at least 10% of the patients. Biguanides (primarily metformin), proton pump inhibitors, NSAIDs, SSRIs, and HMG CoA Reductase Inhibitors were prescribed for at least one-third of the patients (Table 3). Opioid combinations, salicylates, ACE inhibitors, beta blockers cardio-selective, sympathomimetics, loop diuretics, thyroid hormones, insulin, thiazides and thiazide-like diuretics were prescribed for at least 15% of the patients. The median number of medications per patient was 11 with polypharmacy of more than 20 medications in some patients (Additional file 1: Figure S2).

**Table 3:** Use of common medication subclasses (N=2028)

**Medication subclass**	**Frequency**
Biguanides	817 (40%)
Proton Pump Inhibitors	765 (38%)
NSAIDs	765 (38%)
SSRIs	698 (34%)
HMG CoA Reductase Inhibitors	661 (33%)
Opioid Combinations	567 (28%)
Salicylates	533 (26%)
ACE Inhibitors	532 (26%)
Beta Blockers Cardio-Selective	516 (25%)
Sympathomimetics	503 (25%)
Loop Diuretics	452 (22%)
Thyroid Hormones	407 (20%)
Insulin	298 (15%)
Thiazides and Thiazide-Like Diuretics	295 (15%)
Antihypertensive Combinations	263 (13%)
Insulin Sensitizing Agents	258 (13%)
Sulfonylureas	253 (13%)

### Laboratory results

A total of 873 different laboratory results were obtained in the pre-surgery period (Additional file 1: Table S3), with 190 occurring in at least 10% of the patients. Results obtained in the pre-RYGB period for the following laboratory tests were found for at least 95% of the patients: glucose, creatinine, potassium, blood urea nitrogen (bun), calcium, chloride, CO_2_, sodium, hematocrit (hct), hemoglobin (hgb), mean cell hemoglobin (mch), mean cell hemoglobin concentration (mchc), mean cell volume (mcv), platelet count, red blood cell count (rbc), red cell distribution width (rdw), white blood cell count (wbc), estimated glomeruler filtration rate (gfr estimated), albumin, alanine aminotransferase (alt), total protein (protein), alkaline phosphatase, aspartate aminotransferase (ast), total bilirubin, mean platelet volume (mpv), total cholesterol, hdl cholesterol (hdl), triglycerides, cholesterol/hdl ratio, low density lipoprotein (ldl calculated), thyroid stimulating hormone (TSH), hemoglobin A1c, and insulin. Other test results were found for at least half of the patients including ferritin, iron, iron binding capacity, transferrin saturation, anion gap, folic acid, intact parathyroid hormone (PTH), 25 oh vitamin D2 (25 oh D2), 25 oh vitamin D3 (25 oh D3), total vitamin D, urine creatinine, urine protein, urine protein/creatinine ratio, and zinc. Many of the most commonly ordered tests (results in Additional file 1: Table S4) were also ordered following surgery (Table 4), although the post-RYGB period is characterized by clinical need for disease-specific monitoring.

**Table 4:** Availability of common laboratory test results (N=2028)

**Analyte**	**Pre-surgery**	**Post-surgery**
Glucose	99%	92%
BUN	99%	92%
Sodium	99%	92%
Potassium	99%	92%
Chloride	99%	92%
CO2	99%	92%
Creatinine	99%	92%
Calcium	99%	92%
WBC	99%	90%
RBC	99%	90%
Hemoglobin	99%	90%
Hematocrit	99%	90%
MCV	99%	90%
MCH	99%	90%
MCHC	99%	90%
RDW	99%	90%
Platelet count	99%	90%
MPV	99%	90%
ALT	99%	69%
AST	99%	69%
Alkaline phosphate	99%	69%
Total Bilirubin	99%	69%
GFR (estimated)	98%	92%
TSH	98%	37%
Triglycerides	98%	41%
Cholesterol	98%	43%
HDL	98%	41%
LDL	97%	44%
HbA1c	96%	42%
Insulin	95%	14%
Ferritin	88%	52%
Iron	88%	50%
Iron Binding Capacity	88%	48%
Transferrin Saturation	88%	45%
Anion Gap	87%	91%
PTH	83%	71%
Folic Acid	68%	23%

### Survey data

A total of 7 surveys were obtained at some point during the pre- and post-operative periods (Additional file 1: Table S5). When limiting to the subset of patients that were eligible to complete the survey, there were high response rates for the Beck Depression Inventory (BDI, 79% completion), Family Emotional Involvement and Criticism Scale (FEICS, 80% completion), and Impact Of Weight On Quality Of Life-Lite (IWQOL, 70% completion) prior to surgery. The response rates were lower for the remaining surveys, with a 42% completion rate for Weight Loss Readiness, the Sleep Scale, and the work limitations surveys. When limiting to those that were at least one-year post surgery and completed a survey prior to surgery, the number of surveys obtained in the post-operative period was 33% for the BDI and 23% for the IWQOL.

### Length of follow-up

**Figure 2:**
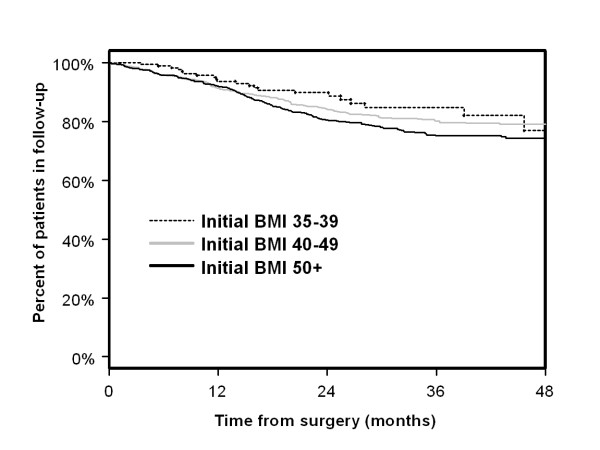
**Kaplan-Meier curves of length of follow-up (N = 2028).** The plots are based upon a sample size of 174 patients with 3255 weight measurements for initial BMI of 35–39 kg/m2, 1032 patients with 20,284 weight measurements for initial BMI of 40–49 kg/m2, and 822 patients with 16,226 weight measurements for BMI of 50+ kg/m2.

The amount of time a patient is followed after a weight loss intervention is extremely important since the percentage of patients that experience weight gain recidivism is high. We used date of surgery and dates associated with follow-up BMI measurements to calculate the percentage of patients lost to follow-up over time using Kaplan-Meier curves (Figure 2 and Additional file 1: Table S6). Because patients with lower degrees of obesity may achieve desired weight loss goals sooner than patients with more extreme levels of obesity [17,18], we stratified patients into three BMI ranges, 35–39, 40–49, and 50+ kg/m^2^. As can be seen in Figure 2, patients in the 35–39 kg/m^2^ range had similar or higher rates of follow-up data until 48 months when they had a 77% follow-up rate versus 79% and 74% for the other two groups (log rank p-value = 0.077), although the sample size is small for the lighter patients. At 1 year all three BMI groups had 92% or greater rates of patients with follow-up data, and at both 24 and 36 months only patients in the highest BMI category were less than 80% (75%). Overall rates of follow-up dropped less than 10% per year over the course of the follow-up period.

We found no significant differences among year of surgery in length of follow-up for 2004, 2005, 2006, and 2007 (log-rank p-value = 0.262). The effect of surgeon on follow-up was also evaluated. There were five primary surgeons that accounted for 96% of the surgeries, with 2 of the 5 who accounted for 66% of the surgeries. These two surgeons were compared with each other and with the other surgeons as a single group. No significant difference was found in length of follow-up (p = 0.130). We also analyzed the type of surgical approach (open versus laparoscopic), which was also not related to length of follow-up (p = 0.123).

## Discussion

The EHR is a longitudinal electronic record of patient health information generated by clinical encounters in a variety of care delivery settings and includes patient demographics, progress notes, problem lists, medications, vital signs, past medical history, immunizations, laboratory data, and radiology reports. EHRs make use of relational database structures and utilities to access and display data that facilitate medical care and clinical decision-making, substituting for traditional paper-based “charts”. EHRs may also help address the long-standing problem of the long lag time that exists before evidence-based medical knowledge is used in clinical care [19]. In addition, the EHR is a robust source of data that can be exploited for research. It provides a means to characterize patients through exploiting existing clinical data, rather than re-capitulating such data via research activities.

In research protocols, physical measures such as height and weight are generally collected by research assistants trained in specific protocols. The tools for those measurements are typically calibrated instruments to reduce error. In contrast, clinical measurements that are recorded in the EHR may be taken by a variety of health care professionals whose procedures may be less stringent, and with equipment that may differ by location. For example, weights may be fully clothed or gowned; heights may be with shoes or without. Some of this type of error can be mitigated by large sample sizes, but some systematic errors cannot. If, for example, the majority of heights are measured with shoes, BMI calculations will be systematically biased downwards. In this study, key height and weight data were extracted from the EHR from measurements obtained in the Weight Management clinic in which a research-like standardized process using calibrated instruments was performed by trained personnel in the same clinic. This standardization also improves the rate of measurement of height, which is measured less frequently than the measurement of weight in EHRs [20].

Data on medication usage and co-morbidities may also present potential problems when extracting from EHRs. We used medication reconciliation and not physician medication orders, sometimes used as a proxy for medication usage. However, not all filled prescriptions will be used by patients. International Classification of Diseases, ninth revision, (ICD-9) codes, which are used for billing, insurance, and documentation in EHRs, as well as for tabulation of clinical statistics and quality analyses, were used as surrogates for the presence of co-morbid conditions. We did not attempt to document the accuracy of individual ICD-9 codes using supporting data such as laboratory results or medication orders. However, the percentages of patients assigned specific ICD-9 codes was similar to the percentage of patients prescribed corresponding medications for a number of major disorders including diabetes and biguanides, depression and SSRIs, and osteoarthosis and NSAIDs. Less closely correlated were hypertension and hypertension medications likely because of the use of tailored polypharmacy to treat individual patients. In addition, the percentage of patients prescribed proton pump inhibitors was higher than the number with GERD likely because of prescribing for other gastrointestinal disorders.

Laboratory data are among the most robust data available from EHRs. We were able to extract a large number of laboratory variables on most patients. Two major reasons for why patients lacked data were that the tests were either never ordered or were obtained at an outside laboratory and the results transmitted in non-electronic form, i.e. paper or scanned portable document format (pdf) copy. This contrasts with survey data in which surveys were offered by providers to each patient, whether in scannable paper format or through on-line access. Since they were not “prescribed” through formal physician order entry, the relative rate of return of this data was less than other types of data domains. Nevertheless, substantial numbers of several survey instruments were obtained.

Despite the presence of clinical data in EHRs that are readily accessed by providers on individual patients, such data can be logistically difficult to extract for research use. Some research groups have been developing natural language processing approaches to obesity-related EHR data [21-23]. We used a data warehouse that mirrored the EHR and greatly facilitated practical access to data. Other obesity research studies have utilized data warehouses [24]. The Veterans Health Administration stores height and weight measurements that have been entered into the EHR in the national Corporate Data Warehouse (CDW). Similar to the Geisinger CDIS data warehouse, the CDW was developed to allow access to data and tools for several purposes including research. Although weight, height and other data are deposited at frequent intervals, the CDW does not yet contain data on laboratory measures, procedures, and diagnoses.

Consensus guidelines have been recommended for determining weight loss following bariatric surgery using operative weight and follow-up weights [25], including the need to define operative weight as the weight at admission or just before surgery. Our data was based upon weights obtained the day of admission based upon the clinic code used for admitting patients for RYGB surgery. Our length of follow-up was also above the recommended minimum rate of 61%.

## Conclusions

EHR data can be a valuable source of data for obesity research, although the availability and integrity of different data types can vary substantially. Access to a data warehouse can greatly enhance the efficiency of data collection over direct extraction from the EHR. EHR derived data can be used for a variety of research and clinical uses such as for determining length of follow-up.

## Competing interest

Co-author Christopher Still receives grant and consulting support from Ethicon-Endosurgery. Co-author Anthony Petrick receives educational grants from Covidien and Ethicon-Endosurgery. Other authors declare no conflict of interest.

## Authors’ contributions

GCW, GSG, PB, GA, and CDS designed the study, directed the data analysis, performed data analysis, and contributed to writing the paper. XC, CM, and JS prepared the data set for analysis. WS, AP, JG, and DC directed the data analysis and contributed to writing the paper. All of the authors have read and approved the final manuscript.

## Pre-publication history

The pre-publication history for this paper can be accessed here:

http://www.biomedcentral.com/1472-6947/12/45/prepub

## Supplementary Material

Additional file 1 Supplementary materials. Click here for file
