# Supplementary material for: An electronic health record-enabled obesity database
Source: BMC Med Inform Decis Mak. 2012 May 28;12:45. doi: 10.1186/1472-6947-12-45 (PMC3508953; doi:10.1186/1472-6947-12-45)

**SUPPLEMENTARY MATERIALS**

**An electronic health record-enabled obesity database.**

**Wood, et al.**

The **Supplementary results** have the following sections in order:

1. **Supplementary Table 1.** List of ICD-9 codes extracted from the EHR.

2. **Supplementary Table 2.** List of medication codes extracted from the EHR.

3. **Supplementary Table 3.** List of Iaboratory results extracted from the EHR.

4. **Supplementary Table 4:** Results of common laboratory tests in the pre-RYGB period.

5. **Supplementary Table 5.** Surveys completed during the Pre- and Post-RYGB periods.

5. **Supplementary Table 6.** Kaplan-Meier table for follow-up at 12, 24, 26, and 48 months post RYGB surgery.

6. **Supplementary Figure 1.** Histogram of number of 5-digit ICD9 codes per patient.

7. **Supplementary Figure 2.** Histogram of number of medication subclasses per patient.

**Supplementary Table 1.** List of ICD-9 codes extracted from the EHR found in more than 100 patients.

| ICD 9 Code | Number of Patients with Code |
| --- | --- |
| 579.3 INTEST POSTOP NONABSORB | 1864 |
| 278.01 MORBID OBESITY, BMI NOT KNOWN | 1796 |
| EP888 ADVANCE DIRECTIVE INFORMATION | 1738 |
| 564.2 POSTGASTRIC SURGERY SYNDROMES | 1011 |
| 250.00 DM TYPE 2, GOAL A1C BELOW 7 | 767 |
| 401.1 HTN, GOAL BELOW 140/90 | 745 |
| 401.9 HTN, GOAL TO BE DETERMINED | 662 |
| 311 DEPRESSIVE DISORDER NEC | 656 |
| 780.57 SLEEP APNEA, UNSPECIFIED | 558 |
| 401.1 HTN, GOAL BELOW 130/80 | 520 |
| 272.4 DYSLIPIDEMIA, GOAL TO BE DETERMINED | 462 |
| 272.0 DYSLIPIDEMIA, GOAL LDL BELOW 100 | 433 |
| 278.00 OBESITY, BMI 30-34 (SEE ACTUAL BMI) | 432 |
| 272.0 DYSLIPIDEMIA, GOAL LDL BELOW 160 | 425 |
| V85.4 OBESITY, BMI 40 AND OVER | 407 |
| 278.00 OBESITY, BMI NOT KNOWN | 363 |
| 278.02 OVERWEIGHT, BMI 25-29 | 361 |
| 250.02 DM TYPE 2, NOT AT GOAL | 359 |
| 244.9 HYPOTHYROIDISM NOS | 340 |
| V67.00 FOLLOWING SURGERY, UNSPECIFIED | 322 |
| 530.10 ESOPHAGITIS, UNSPECIFIED | 304 |
| 729.30 PANNICULITIS, UNSP SITE | 249 |
| 530.81 ESOPHAGEAL REFLUX | 205 |
| 278.01 BMI 35-39 W/ COMORBIDITY (SEE ACTUAL BMI) | 194 |
| 715.98 OSTEOARTHRO NOS-OTH SITE | 190 |
| 272.2 MIXED DYSLIPIDEMIA | 154 |
| 715.00 GENERAL OSTEOARTHROSIS | 147 |
| 278.00 BMI 35-39 ISOLATED (SEE ACTUAL BMI) | 144 |
| 305.1 TOBACCO USE DISORDER | 143 |
| 493.90 ASTHMA,UNSP W/O MENT ST ASTHMAT/AC EXACERB | 143 |
| 268.9 VITAMIN D DEFICIENCY NOS | 139 |
| 493.00 EXTRINSIC ASTHMA, UNSPEC | 130 |
| 251.1 HYPERINSULINISM NEC | 123 |
| 477.9 ALLERGIC RHINITIS NOS | 118 |
| 530.81 GERD | 110 |
| 553.21 INCISIONAL HERNIA | 107 |
| V15.82 HISTORY OF TOBACCO USE | 105 |
| 780.50 SLEEP DISTURBANCE NOS | 104 |

**Supplementary Table 2.** List of medication codes extracted from the EHR found in more than 100 patients..

| Medication Class | Number of Patients |
| --- | --- |
| Selective Serotonin Reuptake Inhibitors (SSRIs) | 569 |
| Biguanides | 540 |
| Nonsteroidal Anti-inflammatory Agents (NSAIDs) | 522 |
| Proton Pump Inhibitors | 518 |
| HMG CoA Reductase Inhibitors | 500 |
| ACE Inhibitors | 418 |
| Salicylates | 411 |
| Sympathomimetics | 376 |
| Loop Diuretics | 348 |
| Beta Blockers Cardio-Selective | 340 |
| Opioid Combinations | 330 |
| Thyroid Hormones | 322 |
| Insulin | 254 |
| Multivitamins | 224 |
| Insulin Sensitizing Agents | 218 |
| Sulfonylureas | 211 |
| Thiazides and Thiazide-Like Diuretics | 208 |
| Antihypertensive Combinations | 203 |
| Serotonin-Norepinephrine Reuptake Inhibitors (SNRIs) | 200 |
| Antihistamines - Non-Sedating | 197 |
| Nasal Steroids | 185 |
| Benzodiazepines | 180 |
| Anticonvulsants - Misc. | 173 |
| Calcium | 171 |
| Calcium Channel Blockers | 168 |
| Angiotensin II Receptor Antagonists | 166 |
| Central Muscle Relaxants | 162 |
| Diagnostic Tests | 162 |
| Potassium | 145 |
| Diabetic Supplies | 115 |
| H-2 Antagonists | 115 |
| Multiple Vitamins w/ Minerals | 113 |
| Opioid Agonists | 112 |
| Leukotriene Modulators | 110 |
| Antidepressants - Misc. | 107 |
| Oil Soluble Vitamins | 104 |

**Supplementary Table 3.** List of Iaboratory results extracted from the EHR found in more than 100 patients..

| Laboratory Test | Number of Patients |
| --- | --- |
| GLUCOSE | 2011 |
| CREATININE | 2010 |
| POTASSIUM | 2010 |
| BUN | 2008 |
| CALCIUM | 2008 |
| CHLORIDE | 2008 |
| CO2 | 2008 |
| SODIUM | 2008 |
| HCT | 1987 |
| HGB | 1987 |
| MCH | 1983 |
| MCHC | 1983 |
| MCV | 1983 |
| PLATELET COUNT | 1983 |
| RBC | 1983 |
| RDW | 1983 |
| WBC | 1983 |
| GFR ESTIMATED | 1981 |
| ALBUMIN | 1977 |
| ALT | 1976 |
| PROTEIN | 1976 |
| ALKALINE PHOSPHATASE | 1975 |
| AST | 1975 |
| BILIRUBIN, TOTAL | 1975 |
| MPV | 1972 |
| CHOLESTEROL | 1967 |
| HDL | 1963 |
| TRIGLYCERIDES | 1962 |
| CHOL/HDL RATIO | 1961 |
| HOURS FASTING | 1961 |
| LDL (CALCULATED) | 1961 |
| TSH | 1955 |
| HEMOGLOBIN, A1C | 1920 |
| INSULIN | 1920 |
| SOURCE | 1792 |
| RESULT/COMMENT | 1785 |
| FERRITIN | 1777 |
| IRON | 1777 |
| IRON BINDING CAP | 1776 |
| TRANSFERRIN SAT % | 1773 |
| ANION GAP | 1768 |
| FOLIC ACID | 1763 |
| PTH, INTACT | 1682 |
| VITAMIN D 25 OH D2 | 1370 |
| VITAMIN D 25 OH D3 | 1370 |
| VITAMIN D 25 TOTAL | 1370 |
| CREATININE, RD URINE | 1199 |
| PROTEIN, RD URINE | 1134 |
| PROT/CREAT RATIO | 1130 |
| ZINC LEVEL | 1067 |
| ABO/RH(D) | 680 |
| ZINC, SERUM | 626 |
| ANTIBODY SCREEN | 608 |
| EST AVG GLUCOSE | 531 |
| LYMPHS | 524 |
| MONOS | 524 |
| SEGS | 524 |
| ABS. LYMPHS | 519 |
| ABS. MONOS | 519 |
| ABS. SEGS | 519 |
| EOS | 511 |
| ABS. EOS | 506 |
| BASOS | 495 |
| RBC MORPH | 492 |
| ABS. BASOS | 490 |
| BILIRUBIN, UA | 221 |
| BLOOD, UA | 221 |
| CLARITY, UA | 221 |
| COLOR, UA | 221 |
| ESTERASE, UA | 221 |
| GLUCOSE, UA | 221 |
| KETONE, UA | 221 |
| NITRITE, UA | 221 |
| PH, UA | 221 |
| PROTEIN, UA | 221 |
| SPECIFIC GRAVITY | 221 |
| UROBILINOGEN, UA | 220 |
| PT/INR-INR | 219 |
| PT/INR-PT | 219 |
| REPORT STATUS | 193 |
| SPECIMEN DESCRIPTION | 193 |
| 25OH VITAMIN D TOTAL | 190 |
| CULTURE | 186 |
| BILIRUBIN, DIRECT | 177 |
| FIO2 | 153 |
| HCG, BETA | 153 |
| O2 FLOW | 153 |
| BICARBONATE, ART | 152 |
| FO2HB, ARTERIAL | 152 |
| O2 SAT, ARTERIAL | 152 |
| PCO2, ARTERIAL | 152 |
| PH, ARTERIAL | 152 |
| PO2, ARTERIAL | 152 |
| PULSE OX | 152 |
| SO2-FO2HB | 152 |
| TEMP, CENTIGRADE | 152 |
| APTT-PATIENT | 151 |
| ALBUMIN, RD URINE | 150 |
| MICROALBUMIN RATIO | 150 |
| HEMOGLOBIN, MEASURED | 134 |
| COTININE | 131 |
| NICOTINE | 131 |
| CARBOXYHEMOGLOBIN | 127 |
| METHEMOGLOBIN | 123 |
| BASE EXCESS, ART | 119 |
| 25 OH VITAMIN D | 111 |
| URIC ACID | 110 |
| COMMENT, UA | 108 |
| GLUCOSE METER | 108 |
| T4, FREE | 105 |
| LDL (DIRECT MEASURE) | 102 |

Supplemental Table 4. Results of common laboratory tests in the pre-RYGB period.

| Analyte | Median | [Q1, Q3] |
| --- | --- | --- |
| Glucose | 95 | [86, 111] |
| BUN | 14 | [12, 18] |
| Sodium | 139 | [138, 141] |
| Potassium | 4.3 | [4.1, 4.5] |
| Chloride | 102 | [100, 104] |
| CO2 | 28 | [26, 29] |
| Creatinine | 0.8 | [0.7, 0.9] |
| Calcium | 9.5 | [9.2, 9.8] |
| WBC | 7.73 | [6.48, 9.29] |
| RBC | 4.58 | [4.33, 4.84] |
| Hemoglobin | 13.6 | [12.9, 14.4] |
| Hematocrit | 39.7 | [37.6, 41.9] |
| MCV | 87.1 | [84.1, 89.9] |
| MCH | 29.9 | [28.7, 30.9] |
| MCHC | 34.3 | [33.8, 34.7] |
| RDW | 13.6 | [13.1, 14.4] |
| Platelet count | 277 | [237, 324] |
| MPV | 8.5 | [7.9, 9.2] |
| ALT | 25 | [19, 35] |
| AST | 23 | [19, 30] |
| Alkaline phosphate | 77 | [65, 92] |
| Total Bilirubin | 0.4 | [0.3, 0.5] |
| GFR (estimated) | >60 | [>60, >60] |
| TSH | 1.935 | [1.31, 2.88] |
| Triglycerides | 147 | [107, 202] |
| Cholesterol | 182 | [160, 210] |
| HDL | 45 | [39, 53] |
| LDL | 103 | [83, 128] |
| HbA1c | 6 | [5.5, 6.7] |
| Insulin | 18.5 | [12.3, 29.3] |
| Ferritin | 88.9 | [46.6, 157.2] |
| Iron | 65 | [51, 82] |
| Iron Binding Capacity | 318 | [289, 351] |
| Transferrin Saturation | 20 | [16, 26] |
| Anion Gap | 10 | [8, 11] |
| PTH | 44 | [33, 58] |
| Folic Acid | 13 | [10.4, 15.7] |

*Q1= first quartile; Q2= third quartile

Supplemental Table 5. Surveys completed during the Pre- and Post-RYGB periods.

| SURVEY NAME | ANY (PRE OR POST) | ANY PRE | ANY POST | PRE AND POST |
| --- | --- | --- | --- | --- |
| BDI | 1792 | 1564 | 633 | 405 |
| FEICS | 1592 | 1398 | 199 | 5 |
| IWQOL | 1553 | 1381 | 426 | 254 |
| WEIGHT LOSS READINESS | 735 | 722 | 15 | 2 |
| SLEEP SCALE | 1011 | 718 | 380 | 87 |
| WORK .LIMITATIONS | 988 | 715 | 344 | 71 |
| QEWP | 1592 | 1398 | 199 | 5 |

Supplemental Table 6. Kaplan-Meier table for follow-up at 12, 24, 26, and 48 months post RYGB surgery.

| BMI (kg/m^2^) | Number | 0 | 12 | 24 | 36 | 48 |
| --- | --- | --- | --- | --- | --- | --- |
| 35-39 | KM estimate  Number at risk | 100%  174 | 94%  136 | 90%  81 | 85%  39 | 77%  10 |
| 40-49 | KM estimate  Number at risk | 100%  1032 | 91%  799 | 84%  503 | 80%  331 | 79%  183 |
| 50+ | KM estimate  Number at risk | 100%  822 | 92%  652 | 80%  434 | 75%  280 | 74%  164 |

Figure 1- Histogram of number of 5-digit ICD9 codes per patient on the active problem list prior to surgery (N=2028).


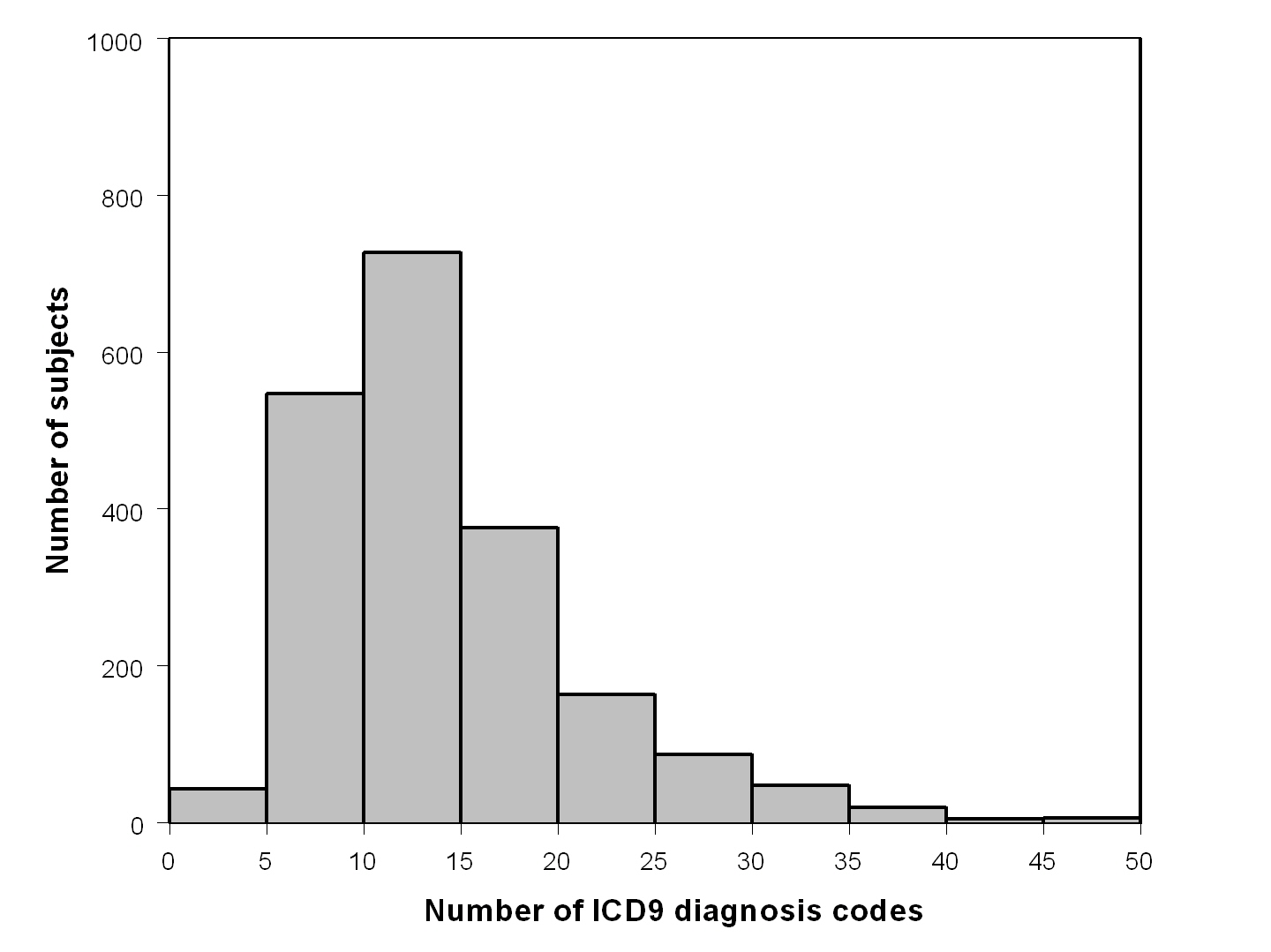


Figure 2. Histogram of number of medication subclasses per patient on the active medication list prior to surgery (N=2028).

.


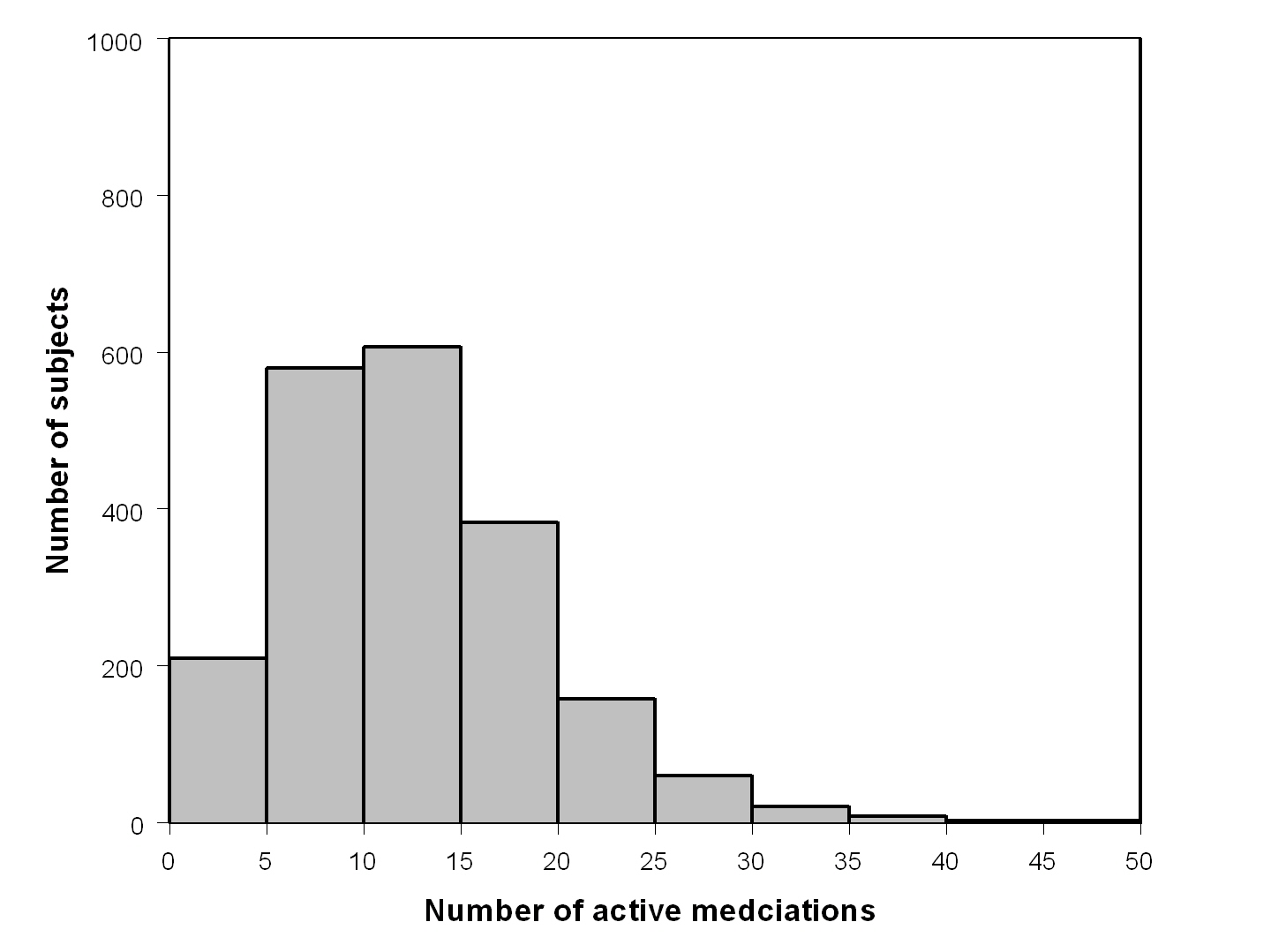

Supplement: Additional file 1 — Supplementary materials. [file 1472-6947-12-45-S1.doc]
